# Variable performance of models for predicting methicillin-resistant *Staphylococcus aureus* carriage in European surgical wards

**DOI:** 10.1186/s12879-015-0834-y

**Published:** 2015-02-27

**Authors:** Andie S Lee, Angelo Pan, Stephan Harbarth, Andrea Patroni, Annie Chalfine, George L Daikos, Silvia Garilli, José Antonio Martínez, Ben S Cooper, in collaboration with the MOSAR-04 Study Team

**Affiliations:** Infection Control Program, University of Geneva Hospitals and Faculty of Medicine, Geneva, Switzerland; Departments of Infectious Diseases and Microbiology, Royal Prince Alfred Hospital, Sydney, Australia; Infectious and Tropical Diseases Unit, Istituti Ospitalieri di Cremona, Cremona, Italy; General Medicine Unit, Ospedale di Esine, Esine, Italy; Infection Control Unit, Groupe Hospitalier Paris Saint-Joseph, Paris, France; First Department of Propaedeutic Medicine, Laiko General Hospital, Athens, Greece; Service of Infectious Diseases, Hospital Clinic de Barcelona, Barcelona, Spain; Mahidol-Oxford Tropical Medicine Research Unit, Faculty of Tropical Medicine, Mahidol University, Bangkok, Thailand; Centre for Clinical Vaccinology and Tropical Medicine, Nuffield Department of Clinical Medicine, University of Oxford, Oxford, UK

**Keywords:** Methicillin-resistant *Staphylococcus aureus*, Screening, Predictive models

## Abstract

**Background:**

Predictive models to identify unknown methicillin-resistant *Staphylococcus aureus* (MRSA) carriage on admission may optimise targeted MRSA screening and efficient use of resources. However, common approaches to model selection can result in overconfident estimates and poor predictive performance. We aimed to compare the performance of various models to predict previously unknown MRSA carriage on admission to surgical wards.

**Methods:**

The study analysed data collected during a prospective cohort study which enrolled consecutive adult patients admitted to 13 surgical wards in 4 European hospitals. The participating hospitals were located in Athens (Greece), Barcelona (Spain), Cremona (Italy) and Paris (France). Universal admission MRSA screening was performed in the surgical wards. Data regarding demographic characteristics and potential risk factors for MRSA carriage were prospectively collected during the study period. Four logistic regression models were used to predict probabilities of unknown MRSA carriage using risk factor data: “Stepwise” (variables selected by backward elimination); “Best BMA” (model with highest posterior probability using Bayesian model averaging which accounts for uncertainty in model choice); “BMA” (average of all models selected with BMA); and “Simple” (model including variables selected >50% of the time by both Stepwise and BMA approaches applied to repeated random sub-samples of 50% of the data). To assess model performance, cross-validation against data not used for model fitting was conducted and net reclassification improvement (NRI) was calculated.

**Results:**

Of 2,901 patients enrolled, 111 (3.8%) were newly identified MRSA carriers. Recent hospitalisation and presence of a wound/ulcer were significantly associated with MRSA carriage in all models. While all models demonstrated limited predictive ability (mean *c*-statistics <0.7) the Simple model consistently detected more MRSA-positive individuals despite screening fewer patients than the Stepwise model. Moreover, the Simple model improved reclassification of patients into appropriate risk strata compared with the Stepwise model (NRI 6.6%, *P* = .07).

**Conclusions:**

Though commonly used, models developed using stepwise variable selection can have relatively poor predictive value. When developing MRSA risk indices, simpler models, which account for uncertainty in model selection, may better stratify patients’ risk of unknown MRSA carriage.

## Background

Mandatory universal methicillin-resistant *Staphylococcus aureus* (MRSA) screening has been introduced in healthcare facilities in many countries, including parts of Europe [1,2]. Recent evidence, however, demonstrates that this approach is not cost-effective in low prevalence settings [3-6] and all European countries have now abandoned mandatory universal MRSA screening. Instead, targeted screening has been advocated. The success of this strategy, however, relies on the development of well-validated MRSA risk indices to guide selection of patients for screening. This is particularly important in surgical wards where appropriate use of perioperative prophylaxis and decolonisation for MRSA carriers may significantly reduce surgical site infections [7-9].

Statistical models have been used to develop risk prediction systems to identify potential MRSA carriers for screening [10-17]. Despite their widespread use, common approaches to prediction modelling, such as stepwise regression, ignore the uncertainty in model selection which can result in overconfident estimates and poor predictive accuracy [18,19]. These limitations may be addressed with other variable selection methods such as model averaging [19]. To our knowledge, no studies have reported the clinical implications of using different model selection techniques for developing MRSA risk indices.

We aimed to compare the performance of various models to predict previously unknown MRSA carriage on admission to surgical wards in 4 European hospitals. We hypothesised that the predictive ability of the commonly-used multivariable stepwise logistic regression method is inferior to other approaches, such as Bayesian model averaging, which account for model uncertainty.

## Methods

### Study design and setting

This study analysed data collected from a prospective cohort study conducted in 13 surgical wards of 4 hospitals in 4 European countries (Athens, Greece; Barcelona, Spain; Cremona, Italy; and Paris, France) [20]. The enrolled wards included vascular (4), orthopaedic (4), cardiac (2), general (2) and neurosurgery (1) subspecialties. The number of annual admissions to the surgical wards at each hospital ranged from 2,723 in Paris to 3,932 in Cremona, with the annual number of surgical procedures ranging from 1,792 in Athens to 3,083 in Paris. Universal MRSA screening on admission was performed on these wards between December 2008 and January 2010. Consecutive adult patients admitted to these wards during this time were eligible for study enrolment. Patients were excluded if they were admitted for less than 24 hours or not screened within 48 hours of admission. Patients already known to be MRSA colonised or infected were also excluded as we were interested in risk profiling patients to identify previously unknown MRSA carriers.

### MRSA screening

MRSA screening swabs were collected from the nose, perineum and wounds if present. Swabs were inoculated directly onto an MRSA chromogenic medium (BBL CHROMagar MRSA II, BD Diagnostics, Belgium) as well as being placed in an enrichment broth to increase the sensitivity of MRSA detection [21]. After incubation at 37°C for 20–24 hours, any characteristically coloured colonies on the chromogenic media were subcultured onto blood agar and incubated overnight. The tube coagulase test, mannitol salt agar subculture and/or the latex agglutination test were used to confirm isolates as *S. aureus*. The enrichment broth, after overnight incubation, was inoculated onto MRSA chromogenic medium if the results of direct plating were negative or indeterminate, then the same procedure as for direct plating was followed.

Screening specimens were processed in local microbiology laboratories which participated in an external quality assurance program [22]. Confirmation of MRSA identification and susceptibility testing was also performed at the study’s central laboratory at the University of Antwerp, Belgium.

### Data collection

Data regarding demographic characteristics, comorbidities and potential risk factors for MRSA carriage were collected using a standardised case report form. These data included age, sex, surgical subspecialty, chronic medical conditions, hospitalisation in the last year, surgery in the last 3 months, history of transfer from another ward or healthcare facility, nursing home residency, presence of a skin wound or pressure sore, antibiotic use in the last 6 months and the presence of long-term invasive devices.

The primary outcome of interest was previously unknown MRSA colonisation on admission defined as the detection of MRSA from a screening swab or clinical sample collected within 48 hours of admission to the surgical ward.

### Statistical analysis

Univariable analyses of baseline characteristics were performed using χ^2^ test or Student’s *t*-test as appropriate. For the multivariable analysis, we compared the commonly used stepwise variable selection approach with Bayesian model averaging (BMA) which provides a mechanism for accounting for model uncertainty with the aim of improving prediction accuracy [18,19]. The stepwise approach involved stepwise backward logistic regression with inclusion of all covariates with *P* < .2 in the primary model. Likelihood ratio tests were used to guide exclusion of covariates from the model. Covariates with *P* ≤ .05 were retained in the model. The BMA approach averaged over multiple models and used the posterior probabilities of these models to perform all inferences and predictions [19]. We developed 4 multivariable logistic regression models: 1) “Stepwise” model – which used stepwise backward elimination; 2) “Best BMA” model – which was the model with the highest posterior probability after using the Bayesian model averaging approach; 3) “BMA” model – which included all covariates with a posterior probability greater than zero with Bayesian model averaging; 4) “Simple” model – which included covariates selected at least half the time in both Stepwise and BMA models on repeated random sub-samples of 50% of the data from the whole cohort. The aim of the “Simple” model strategy was to develop a simple or parsimonious model with a clinically meaningful level of prediction with as few predictor variables as possible. All tests were 2-tailed and *P* ≤ .05 was considered statistically significant.

To evaluate the predictive performance of the models, cross-validation was performed using 1000 repeated random sub-samples of the data of the entire cohort divided in a 1:1 ratio into derivation and validation datasets. Models were constructed with the first half of the data (derivation dataset). The results of this analysis were then used to predict unknown MRSA carriage in the other half of the data (validation dataset). The *c*-statistic (or area under the receiver operating characteristic (ROC) curve which plots sensitivity against 1-specificity) was calculated to determine the models’ ability to discriminate between individuals who were and were not MRSA colonised. The *c*-statistic has a theoretical range from 0 to 1, with a statistically meaningful range from 0.5 (no predictive ability) to 1 (perfect discrimination) [23]. A mean *c*-statistic was calculated from the logistic models fitted to the derivation cohorts from the repeated random sub-samples of the data. To assess the agreement between predicted and observed MRSA carriage, the relative frequencies of predicted probabilities (density plots) of MRSA colonisation were plotted by observed MRSA colonisation status as determined by admission screening. Because the derivation and validation datasets are from the same 4 hospitals, the results may overestimate the likely predictive performance when applied to other hospitals. To overcome this problem, models were fitted using data from 3 of 4 hospitals as the derivation dataset with each hospital in turn being used as the validation dataset.

Similarly, the clinical utility of the models was evaluated by selecting a random sample of 50% of the full cohort as the derivation dataset and using the remaining data as the validation dataset. We calculated the sensitivity, specificity, positive predictive value (PPV) and negative predictive value (NPV) for screening patients at varying levels of predicted probabilities of MRSA carriage as determined by the 4 models. We then used a cut-off of 4% predicted probability of MRSA carriage to categorise patients as low- or high-risk using the Stepwise model. The accuracy with which each model stratified risk was compared with that of the Stepwise model by calculating the number of patients who were reclassified into different risk categories using each model [23]. Any upward movement in predicted risk for subjects with MRSA implied improved reclassification, and any downward movement indicated worse reclassification. The opposite applied for MRSA negative subjects. The improvement in reclassification was quantified as the net reclassification improvement (NRI) (sum of differences in proportion of individuals moving up minus the proportion moving down for those with MRSA, and the proportion of individuals moving down minus the proportion moving up for those without MRSA) [24]. The analyses were performed with R statistical software including the BMA package [25,26].

### Ethics statement

The study was approved by the Institutional Review Board of the University of Geneva Hospitals (Comité d’Ethique N.A.C.), the location of the coordinating centre. Local ethics committee approval was also obtained from each participating hospital as a quality improvement project with a waiver of individual informed consent (Institution Review Board of Laiko General Hospital, Athens; Comité Etico de Investigación Clínica, Barcelona; Comitato Etico degli Istituti Ospitalieri di Cremona, Cremona; Comité de Protection des Personnes – Ile-de-France IX, Paris).

## Results

There were 2,935 patients screened on admission to the surgical wards. Among these, 34 (1.2%) patients were excluded as they were previously known to be MRSA colonised or infected. The remaining 2,901 patients were included in the study, of which 111 (3.8%) were newly identified MRSA carriers. Patient characteristics are shown in Table 1. Risk factors for MRSA carriage on admission on univariable analysis included older age, cerebrovascular disease, diabetes mellitus, chronic obstructive pulmonary disease, chronic skin disease, hospitalisation in the last year, nursing home residency, presence of a skin wound or pressure sore, antibiotic use in the last 6 months, urinary catheterisation and presence of a tracheostomy.

**Table 1:** **Characteristics of patients colonised and not colonised with methicillin-resistant** ***Staphylococcus aureus*** **on admission**

**Characteristic**	**MRSA positive**	**MRSA negative**	***P***
	**(** ***n*** **= 111)**	**(** ***n*** **= 2,790)**	
Hospital			<.001
Athens (*n* = 985)	51 (5.2)	934 (94.8)	
Barcelona (*n* = 510)	9 (1.8)	501 (98.2)	
Cremona (*n* = 817)	20 (2.4)	797 (97.6)	
Paris (*n* = 589)	31 (5.3)	558 (94.7)	
Age, mean (SD), years	70.3 (16.5)	64.5 (17.9)	<.001
Female sex	59 (53.2)	1,358 (48.7)	.355
Surgical subspecialty			.112
General	29 (26.1)	639 (22.9)	
Vascular	31 (27.9)	779 (27.9)	
Neurosurgery	7 (6.3)	229 (8.2)	
Orthopaedics	42 (37.8)	910 (32.6)	
Cardiothoracic	2 (1.8)	233 (8.4)	
Comorbidities			
Chronic renal failure	8 (7.2)	131 (4.7)	.224
Haemodialysis	1 (0.9)	27 (1.0)	.944
Cardiovascular disease	69 (62.2)	1,584 (56.8)	.261
Cerebrovascular disease	4 (3.6)	33 (1.2)	.026
Diabetes mellitus	34 (30.6)	471 (16.9)	<.001
COPD	19 (17.1)	273 (9.8)	.012
Solid organ malignancy	18 (16.2)	402 (14.4)	.596
Haematological malignancy	1 (0.9)	10 (0.4)	.362
Autoimmune disease	1 (0.9)	63 (2.3)	.340
Liver cirrhosis	4 (3.6)	58 (2.1)	.276
HIV infection	0 (0.0)	11 (0.4)	.507
Trauma	24 (21.6)	554 (19.9)	.648
Chronic skin disease	12 (10.8)	98 (3.5)	<.001
Recent hospitalisation (<1 year)	60 (54.1)	862 (30.9)	<.001
Recent surgery (<3 months)	17 (15.3)	277 (9.9)	.065
Transfer from another ward or hospital	8 (7.2)	231 (8.3)	.687
Nursing home resident	11 (9.9)	75 (2.7)	<.001
Skin wound/ulcer	26 (23.4)	200 (7.2)	<.001
Recent antibiotic use (<6 months)	47 (42.3)	674 (24.2)	<.001
Indwelling devices			
Long-term vascular catheter	1 (0.9)	22 (0.8)	.896
Urinary catheter	8 (7.2)	42 (1.5)	<.001
Tracheostomy	3 (2.7)	17 (0.6)	.009
Other device	2 (1.8)	53 (1.9)	.941

Note. Data are no. (%) of patients unless otherwise indicated. COPD, chronic obstructive pulmonary disease; HIV, human immunodeficiency virus; MRSA, methicillin-resistant *Staphylococcus aureus*; SD, standard deviation.

### Multivariable models

**Figure 1:**
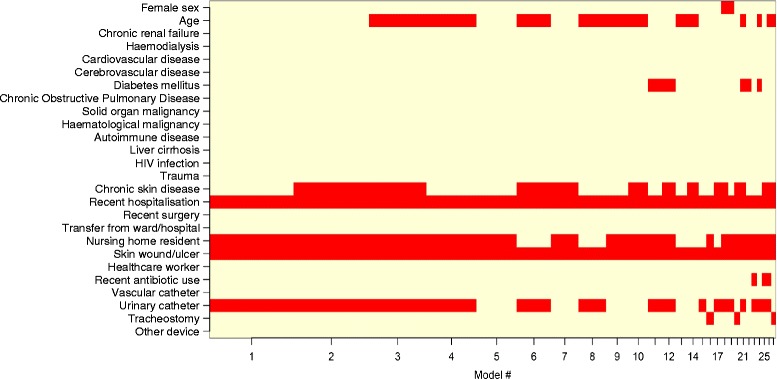
**Methicillin-resistant** ***Staphylococcus aureus*** **predictors selected in various models by Bayesian model averaging.** Note. HIV, human immunodeficiency virus.

Independent predictors of MRSA carriage on admission for each multivariable model are shown in Table 2. Recent hospitalisation and the presence of a wound or pressure sore were significantly associated with MRSA carriage in all models. The Stepwise model identified 6 independent risk factors for MRSA colonisation (older age, chronic skin disease, hospitalisation in the last year, nursing home residency, presence of a wound or pressure sore and urinary catheterisation). The covariates in the first 27 models selected by the BMA approach are illustrated in Figure 1. The Best BMA model, the model with the highest posterior probability (Model #1 in Figure 1), included nursing home residency and urinary catheterisation in addition to the 2 risk factors selected in all models. The BMA model, which averaged over the multiple selected models, only identified the 2 risk factors common to all models as significant but included 10 risk factors in total. In the Simple model, MRSA carriage was associated with 5 covariates (those of the Stepwise model excluding urinary catheterisation).

**Table 2:** **Results of multivariable models of risk factors for methicillin-resistant** ***Staphylococcus aureus*** **carriage on admission**

**Risk factor**	**Stepwise model**			**Best BMA model**			**BMA model**				**Simple model**		
	**OR**	**95% CI**	***P***	**OR**	**95% CI**	***P***	**Posterior Probability**	**OR**	**95% CI**	***P***	**OR**	**95% CI**	***P***
Female sex							2.3	1.0	0.9-1.1	.894			
Age (per 1-year increment)	1.02	1.00-1.03	.009				44.8	1.01	0.99-1.03	.421	1.02	1.00-1.03	.009
Diabetes							7.8	1.0	0.8-1.4	.796			
Chronic skin disease	3.0	1.5-5.8	.002				49.3	1.7	0.5-5.3	.369	2.9	1.5-5.6	.002
Hospitalisation (<1 year)	2.2	1.5-3.3	<.001	2.3	1.5-3.4	<.001	100	2.2	1.5-3.4	<.001	2.2	1.5-3.3	<.001
Nursing home resident	3.4	1.6–6.8	.001	4.2	2.1-8.3	<.001	82.4	3.1	0.9-10.3	.070	3.4	1.7-6.9	.001
Skin wound/sore	2.7	1.7-4.4	<.001	3.0	1.8-4.8	<.001	100	2.9	1.7-4.7	<.001	2.8	1.7-4.6	<.001
Antibiotics (<6 months)							2.6	1.0	0.9-1.2	.890			
Urinary catheter	4.5	2.0-10.3	.018	4.3	1.9-9.6	<.001	72.4	2.9	0.7-12.8	.152			
Tracheostomy							3.1	1.1	0.6-2.0	.867			
*C*-statistics for validation dataset													
Cross-validation, mean (SD)^a^	0.643 (0.029)			0.663 (0.028)				0.653 (0.031)			0.687 (0.030)		
Athens	0.598			0.610				0.606			0.640		
Barcelona	0.762			0.797				0.798			0.797		
Cremona	0.585			0.641				0.601			0.670		
Paris	0.585			0.579				0.627			0.686		

Note. The Stepwise model used stepwise backward elimination; the Best BMA model was the model with the highest posterior probability with the Bayesian model averaging approach; the BMA model included all covariates with a posterior probability of greater than zero using Bayesian model averaging; the Simple model included variables selected at least half the time in both Stepwise and BMA models on repeated random sub-samples of 50% of the cohort. CI, confidence interval; OR, odds ratio; SD, standard deviation.

^a^Cross-validation by repeated random sub-sampling of 50% of the full cohort data for derivation and validation datasets.

### Assessment of the performance of the prediction models

**Figure 2:**
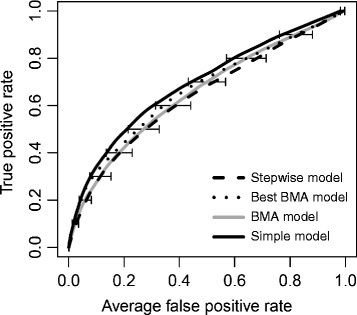
**Receiver operating characteristic (ROC) curves for the multivariable models of admission methicillin-resistant** ***Staphylococcus aureus*** **carriage.** Note. BMA, Bayesian model averaging.

**Figure 3:**
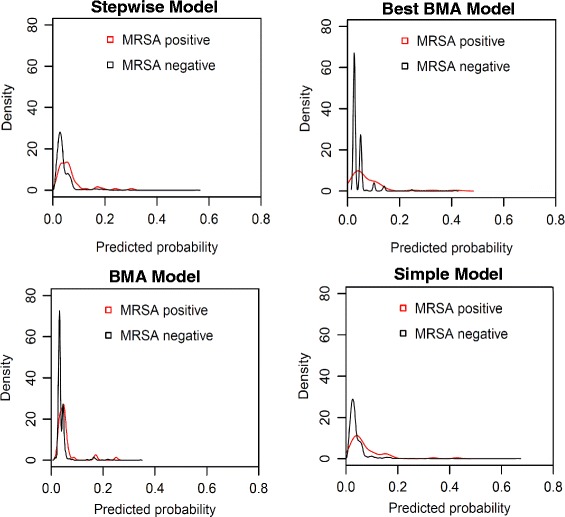
**Density plots showing the relative frequencies of predicted probabilities of admission methicillin-resistant** ***Staphylococcus aureus*** **colonisation.** Note. BMA, Bayesian model averaging.

After cross-validation, the mean *c*-statistics were 0.643 (SD 0.029) for the Stepwise model, 0.663 (SD 0.028) for the Best BMA model, 0.653 (SD 0.031) for the BMA model, and 0.687 (SD 0.030) for the Simple model (Table 2), demonstrating limited ability of all the models to discriminate between patients who were and were not MRSA colonised. The ROC curves for all models were similar, though showed that the Stepwise model consistently had the worst performance and the Simple model was consistently best (Figure 2). The density plots in Figure 3 also show that there is considerable overlap between MRSA positive and negative patients for the range of probabilities of MRSA carriage predicted by the models, particularly for low predicted probabilities.

When each hospital was used in turn as the validation cohort, the Simple model was again most discriminatory in all models (*c*-statistics in Table 2). However, these models performed less well than the models that were fitted to data from the whole cohort. The discrimination of the models was in the poor to limited range (*c*-statistics 0.579 to 0.686) except when the models were validated in the Barcelona cohort where the models had higher *c*-statistic values (0.762 to 0.798).

Table 3 summarises the changes in screening performance with the use of different cut-offs of predicted probability of MRSA carriage. For all cut-offs, the Simple model detected more MRSA-positive patients than the Stepwise model despite screening fewer patients in total, giving an improvement in PPV of up to 15%. Targeted screening of patients with a predicted MRSA carriage risk of greater than or equal to 4% would result in similar reduction in screening volume using all four models (64% to 68%). For this cut-off, the sensitivity for detecting MRSA carriers ranged from 61% to 69%, with the lowest sensitivity for the Stepwise model and the highest for the Best BMA model. The Simple model, however, had the highest PPV, on account of the smaller number of patients screened, while the Stepwise model had the lowest PPV. At higher cut-offs, larger differences between models were seen. For example, with a cut-off of 6%, the Best BMA model would screen 45% fewer patients than the Stepwise model, but detect only one fewer MRSA patient, corresponding to a 70% improvement in the PPV. Due to the relatively low prevalence of MRSA carriage in this cohort (3.8%), all models had high NPVs.

**Table 3:** **Comparison of methicillin-resistant** ***Staphylococcus aureus*** **(MRSA) screening strategies using different predictive models**

**Cut-off for screening**	**No. of patients to be screened (%)**	**No. of patients MRSA positive**	**Sensitivity (%)**	**Specificity (%)**	**PPV (%)**	**NPV (%)**
Universal screening	1,451 (100)	49	100	0	3.4	-
Predicted probability ≥ 2%						
Stepwise model	1,213 (83.6)	46	93.9	16.8	3.8	98.7
Best BMA model	1,451 (100)	49	100	0	3.4	-
BMA model	1,437 (99.0)	49	100	1.0	3.4	100
Simple model	1,201 (82.8)	47	95.9	17.7	3.9	99.2
Predicted probability ≥ 3%						
Stepwise model	813 (56.0)	37	75.5	44.7	4.6	98.1
Best BMA model	528 (36.4)	34	69.4	64.8	6.4	98.4
BMA model	1,137 (78.4)	44	89.8	22.0	3.9	98.4
Simple model	776 (53.5)	39	79.6	47.4	5.0	98.5
Predicted probability ≥ 4%						
Stepwise model	482 (33.2)	30	61.2	67.8	6.2	98.0
Best BMA model	528 (36.4)	34	69.4	64.8	6.4	98.4
BMA model	463 (31.9)	31	63.3	69.2	6.7	98.2
Simple model	479 (33.0)	33	67.3	68.2	6.9	98.4
Predicted probability ≥ 5%						
Stepwise model	354 (24.4)	26	53.1	76.6	7.3	97.9
Best BMA model	167 (11.5)	19	38.8	89.4	11.4	97.7
BMA model	133 (9.2)	16	32.7	91.7	12.0	97.5
Simple model	336 (23.2)	27	55.1	78.0	8.0	98.0
Predicted probability ≥ 6%						
Stepwise model	229 (15.8)	18	36.7	85.0	7.9	97.5
Best BMA model	126 (8.7)	17	34.7	92.2	13.5	97.6
BMA model	64 (4.4)	6	12.2	95.9	9.4	96.9
Simple model	220 (15.2)	20	40.8	85.7	9.1	97.6

Note. The table shows the results when a random sample of 50% of the full cohort was used as the derivation dataset with the remaining data used as the validation dataset. MRSA, methicillin-resistant *Staphylococcus aureus*; NPV, negative predictive value; PPV, positive predictive value.

Using a cut-off of 4% for the predicted probability of MRSA carriage led to the classification of 482 of 1,451 (33.2%) patients in the validation cohort as high-risk with the Stepwise model. Table 4 shows how patients are reclassified into risk groups for each model compared with the Stepwise model for this cut-off. The measure of correct reclassification (NRI) was 5.2% (95% CI −6.3%-16.6%, *P* = .38) for the Best BMA model compared to the Stepwise model, indicating that 5.2% more MRSA positive patients were appropriately moved up a risk category than down compared to MRSA negative patients. The NRIs were 5.6% (95% CI −3.4%-14.6%, *P* = .22) and 6.6% (95% CI −0.5%-13.6%, *P* = .07) for the BMA and Simple models compared to the Stepwise model, respectively.

**Table 4:** **Reclassification in predicted risk of methicillin-resistant** ***Staphylococcus aureus*** **carriage between models**

**Stepwise model**	**Best BMA model**			**BMA model**			**Simple model**		
**Frequency (Row %)**	**<4%**	**≥4%**	**Total**	**<4%**	**≥4%**	**Total**	**<4%**	**≥4%**	**Total**
Patients who screened MRSA positive									
<4%	13 (68.4)	6 (31.6)	19	16 (84.2)	3 (15.8)	19	16 (84.2)	3 (15.8)	19
≥4%	2 (6.7)	28 (93.3)	30	2 (6.7)	28 (93.3)	30	0 (0)	30 (100)	30
Total	15	34	49	18	31	49	16	33	49
Patients who screened MRSA negative									
<4%	852 (89.7)	98 (10.3)	950	917 (96.5)	33 (3.5)	950	915 (96.3)	35 (3.7)	950
≥4%	56 (12.4)	396 (87.6)	452	53 (11.7)	399 (88.3)	452	41 (9.1)	411 (90.9)	452
Total	908	494	1,402	970	432	1,402	956	446	1,402
NRI (95% CI)	5.2% (−6.3%-16.6%) *P* = .376			5.6% (−3.4%-14.6%) *P* = .222			6.6% (−0.5%-13.6%) *P* = .068		

Note. The table shows the results when a random sample of 50% of the full cohort was used as the derivation dataset with the remaining data used as the validation dataset. CI, confidence interval; MRSA, methicillin-resistant *Staphylococcus aureus*; NRI, net reclassification improvement of each model compared with the Stepwise model.

## Discussion

The development of a successful targeted MRSA screening strategy requires a robust tool for identifying individuals at increased risk of MRSA carriage. Prediction models can quantify this risk and therefore facilitate screening of individuals with a predicted risk above a selected threshold, with the ultimate aim of increasing the cost-effectiveness of the screening strategy [12]. We compared different modelling approaches and found that the predictive performance of the models to identify unknown MRSA carriage on admission to surgical wards was limited. However, when we assessed model performance using cross-validation, we were able to demonstrate that the commonly-used stepwise model selection approach has inferior predictive performance to approaches that account for model uncertainty.

There is no consensus on the optimal approach to building a multivariable predictive model [27]. Alternative methods to stepwise variable selection include use of full(er) models which leave non-significant variables in the model, expert knowledge to guide variable selection and automated approaches such as Lasso and Least Angle Regression (LAR) methods [28]. Each approach has its advantages and disadvantages. We specifically set out to compare stepwise logistic regression, as it is widely used in prediction modelling despite its problems, with Bayesian Model Averaging, which can potentially overcome some of the limitations of stepwise approaches. The stepwise strategy ignores the variables which are not selected as well as the uncertainty or imprecision resulting from the variable selection process itself since the final single model is assumed to be “optimal” [19]. Because automated stepwise variable selection procedures generate a model to provide the best fit for the available data, there is the potential that the model will be overfitted and hence provide an optimistic assessment of its predictive ability [29]. This “optimism” results in worse prediction in independent data [30]. In contrast, BMA selects a number of all possible single models and uses their posterior probabilities to perform all inferences with the aim of improving predictive performance [18]. BMA has been shown to be less likely than stepwise regression to select redundant variables while having a similar probability of selecting a true predictor [31]. We found that the modelling approaches that account for model uncertainty out-performed the stepwise strategy when used to predict MRSA carriage risk. Similar findings have been demonstrated in other areas of research such as cardiovascular risk prediction [19].

In our study, targeted screening of patients with predicted probabilities of MRSA carriage of greater than or equal to 4% would reduce screening burden by about two-thirds compared with universal screening using both the Stepwise and Simple models. However, the Simple model was more sensitive, identifying 6.1% more MRSA carriers. The rationale for the Simple model was twofold. First, it accounted for model uncertainty by incorporating the BMA approach in the strategy. It also included covariates that were selected in the majority of models on repeated random sub-samples of the data with the aim of identifying factors that were truly informative since repetition of the procedure allowed more of the data to be used for derivation of the models. Second, it was expected that this approach would result in a more parsimonious model containing fewer covariates as it only included those that were commonly selected by both Stepwise and BMA models. This “simple” model would reduce the risk of overfitting. Indeed, this approach produced the model with the best predictive performance. A “simple” model would also be easier to implement in the clinical setting as the number of variables for which information would need to be collected would be smaller.

The risk of overfitting a model increases if the number of outcomes is small [32]. Thus the performance of predictive models developed from cohorts with high MRSA prevalence would be expected to be superior to that of models based on cohorts with few MRSA positive subjects. Indeed this was demonstrated in our evaluation of model performance where each hospital was used in turn as the validation dataset. The number of MRSA-positive subjects was lowest in the Barcelona cohort in which only 9 (1.8%) patients were MRSA positive compared with 20 to 51 (2.4% to 5.2%) MRSA carriers in the other hospitals. This difference in MRSA prevalence may explain why the models did not perform well, as measured by the *c*-statistic, except when data from Barcelona were used in the validation rather than the derivation dataset. The superior performance of the models to predict MRSA carriage in the Barcelona cohort could also be explained by the relatively homogenous group of MRSA patients in this hospital. Most patients had the “typical” risk factors of older age, recent hospitalisation, nursing home residency and presence of wounds.

We evaluated the clinical implications of using each model compared with the Stepwise model by quantifying the reclassification of patients into appropriate risk groups. Our results showed that a cut-off of 4% for the predicted probability of MRSA carriage would classify 33.2% of patients as high-risk using the Stepwise model. These patients could be targeted for screening. However, compared with the Stepwise model, use of the Simple model would increase appropriate patient screening by approximately 6.6% (*P* = .07). This improvement in reclassification of patients with the Simple model occurred despite little change in the *c*-statistic, demonstrating that the traditionally used *c*-statistic may be an insensitive measure of model performance. Novel measures, such as the NRI, may be more useful for comparing prediction models [23,33]. Once the model is chosen, the risk cut-off selected for the screening algorithm will be a trade-off between the sensitivity and specificity, and where this cut-off is set is an economic decision which should ideally be evaluated using decision models.

We explored risk factors for MRSA carriage in a number of surgical units in different countries using a large sample size, reducing the risk of overfitting the models. Previous studies have limited their analyses to single centres or a number of centres in the same country [10,14,15,17]. In addition, we included subjects who were expected to have varying levels of MRSA carriage risk. The data used in the study was also collected prospectively, increasing the quality of the information on risk factors. We screened at least two anatomic sites for MRSA carriage as well as using overnight enrichment for screening specimens [21,34], increasing the sensitivity of MRSA detection. Screening for MRSA from nasal samples alone or with less sensitive laboratory techniques may underestimate true MRSA carriage rates and lead to differences in results between studies. We attempted to correct for overoptimistic model predictions by cross-validation. In addition, we assessed the performance of the models in different patient groups by using each hospital in turn as the validation set.

Our study has some limitations. The predictive performance of the models may be affected by the emergence of community-associated MRSA which, in some regions, is now the commonest cause of soft tissue infection among persons who have not had healthcare contact [35]. Livestock-related MRSA is also an increasing problem in some countries [36]. These emerging strains of MRSA are not necessarily associated with the traditional risk factors of healthcare-associated strains. However, in the 4 hospitals participating in our study, these new MRSA strains remain relatively rare [37]. It would be important to externally validate our predictive models, using a dataset different in time and place to the one from which it was developed, to determine if their performance properties are maintained in, and therefore generalisable to, different patient populations [38]. Geographic variations in MRSA epidemiology may warrant the development of local prediction rules to increase the accuracy of risk stratification for selection of patients for MRSA screening.

## Conclusions

Multidrug-resistant organisms are an increasing global problem [39]. Risk prediction models which can accurately quantify the probability of carriage with these organisms can assist in targeting active surveillance and control measures, thus increasing the cost-effectiveness of these interventions [40]. It is important to note, however, that the performance of different model selection approaches to develop MRSA risk indices varies. Our study showed that although the predictive performance of the various approaches was limited, simpler or more parsimonious models, which account for model uncertainty, may perform better than commonly-used stepwise models when assessed with novel, clinically relevant performance measures. With emerging evidence to support preoperative interventions to reduce infections in individuals colonised with *S. aureus* [9,41], the development of robust risk profiling tools to identify carriers of both methicillin-sensitive as well as methicillin-resistant *S. aureus* would be an important focus for future research.

## Abbreviations

BMA: Bayesian model averaging
CI: Confidence interval
COPD: Chronic obstructive pulmonary disease
HIV: Human immunodeficiency virus
MRSA: Methicillin-resistant *Staphylococcus aureus*
NPV: Negative predictive value
NRI: Net reclassification improvement
OR: Odds ratio
PPV: Positive predictive value
ROC: Receiver operating characteristic
SD: Standard deviation
